# Indium Incorporation into InGaN Quantum Wells Grown on GaN Narrow Stripes

**DOI:** 10.3390/ma12162583

**Published:** 2019-08-14

**Authors:** Marcin Sarzyński, Ewa Grzanka, Szymon Grzanka, Grzegorz Targowski, Robert Czernecki, Anna Reszka, Vaclav Holy, Shugo Nitta, Zhibin Liu, Hiroshi Amano, Mike Leszczyński

**Affiliations:** 1 Institute of High Pressure Physics PAS, Sokołowska 29/37, 01-142 Warsaw, Poland; 2 TopGaN Ltd., Sokołowska 29/37, 01-142 Warsaw, Poland; 3 Institute of Physics PAS, Al. Lotnikow 32/46, 02-668 Warsaw, Poland; 4 Faculty of Mathematics and Physics, Charles University, Ke Karlovu 5, 121 16 Praha 2, Czech Republic; 5 Institute of Materials and Systems for Sustainability, Nagoya University, Furo-cho, Chikusa-ku, Nagoya 464-8603, Japan; 6 Department of Electrical Engineering and Computer Science, Nagoya University, Furo-cho, Chikusa-ku, Nagoya 464-8603, Japan; 7 Akasaki Research Center, Nagoya University, Furo-cho, Chikusa-ku, Nagoya 464-8603, Japan; 8 Venture Business Laboratory, Nagoya University, Furo-cho, Chikusa-ku, Nagoya 464-8603, Japan

**Keywords:** InGaN, vapor phase epitaxy, patterned substrate, quantum wells, multicolor emitters

## Abstract

InGaN quantum wells were grown using metalorganic chemical vapor phase epitaxy (vertical and horizontal types of reactors) on stripes made on GaN substrate. The stripe width was 5, 10, 20, 50, and 100 µm and their height was 4 and 1 µm. InGaN wells grown on stripes made in the direction perpendicular to the off-cut had a rough morphology and, therefore, this azimuth of stripes was not further explored. InGaN wells grown on the stripes made in the direction parallel to the GaN substrate off-cut had a step-flow-like morphology. For these samples (grown at low temperatures), we found out that the InGaN growth rate was higher for the narrower stripes. The higher growth rate induces a higher indium incorporation and a longer wavelength emission in photoluminescence measurements. This phenomenon is very clear for the 4 µm high stripes and less pronounced for the shallower 1 µm high stripes. The dependence of the emission wavelength on the stripe width paves a way to multicolor emitters.

## 1. Introduction

Most electronic and optoelectronic devices are fabricated on laterally homogeneous epitaxial structures, however, having structures of properties varying in lateral directions would offer new possibilities, for example, monolithic integration of different devices. Such epitaxial structures are prepared by lateral patterning (lithography and masking) and overgrowth.

The first epitaxial lateral overgrowth (ELOG) of silicon and GaAs over SiOx masks was demonstrated almost 40 years ago [1,2,3,4]. This technology was then used for GaN growth on highly mismatched substrates—sapphire [5,6,7], SiC [8], or silicon [9]. In all cases, laterally grown GaN over the mask had substantially lower threading dislocation density than in areas of mask openings. The next step of this lateral overgrowth was pendeo epitaxy on stripes made on the GaN layer on foreign substrate [10]. Also, in that kind of epitaxy, the suspended wings contained much lower dislocations.

Pendeo epitaxy of GaN paved the way to growth of AlGaInN epitaxial structures on laterally pattered substrates. In the case of highly mismatched materials, lateral patterning may not only lead to lateral chemical composition variation, but also to elastic strain relaxation. Having submicron patterning, one may obtain quantum dots and wires [11,12].

In the present paper we focus on the growth of the ternary alloy, InGaN, on patterned GaN substrates. InGaN is the key material in III-Nitride optoelectronic devices such as light emitting diodes (LEDs) and laser diodes. operating in the blue and green ranges of the electromagnetic spectrum. Single and multiple quantum wells (SQWs, MQWs or just QWs for short) made of InGaN usually form the active region of such devices, which are typically grown on different sorts of GaN substrates. However, InGaN is relatively difficult to be grown high-quality (compared to the base binary compound, GaN) because of its large lattice mismatch to GaN 11% [13,14] and low growth temperature—700–800 °C for InGaN [15,16,17] versus 950–1050 °C for GaN. These two factors induce a number of crystallographic defects, such as indium concentration fluctuations [18,19,20,21] and a large concentration of point defects [22]. Such defects, in turn, adversely affect optical properties of devices.

Growth of InGaN on three-dimensional structures has been studied in a number of papers [23,24]. These papers dealt mainly with the growth of nanocolumns. Such three-dimensional growth is possible due to the low atomic incorporation at the side walls, and a high incorporation on the (00.1) surface. InGaN growth on a substrate with stripes was studied by Fang and coworkers [25] who studied the mass transport mechanism in InGaN epitaxy on ridge-shaped selective area growth GaN by metal organic chemical vapor deposition. In that work, however, emphasis was put on different aspects and the authors did not describe indium composition as a function of the stripe width. They also used selective mask for the growth, which is not the case in the present paper.

In one of our previous papers [26] we reported on InGaN quantum wells grown on stripes with various off-cuts. As indium incorporation into InGaN depends on the off-cut [27], by varying the off-cut spatially we could produce monolithically integrated multicolor emitters. However, when decreasing the size of the stripes we found out that not only the substrate off-cut, but also the size of the stripes influences indium incorporation into InGaN. The present paper reports on this phenomenon—we will show that the growth rate depends on the geometric dimensions of the stripes (width and height). Because indium incorporation into InGaN depends on the growth rate [28,29], consequently. In content also changes with the width of the stripes.

## 2. Experimental

### 2.1. Substrate Preparation

**Figure 1:**
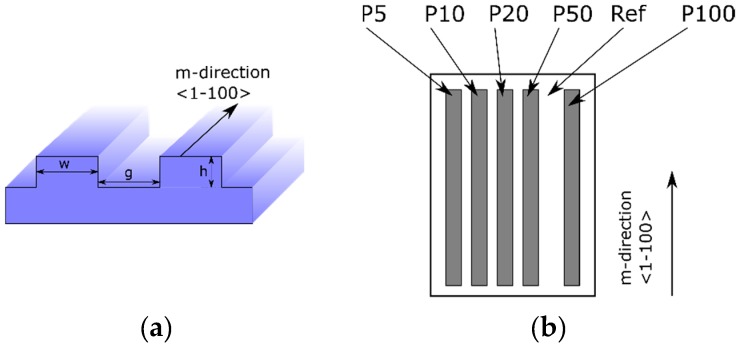
Stripes fabricated in GaN substrates. (**a**) Schematic perspective view and definition of stripe dimensions. (**b**) Scheme of a 11 × 14 mm c-plane (0001) GaN substrate with five patterned regions marked P5, P10, P20, P50, and P100. The stripe width (w) is 5, 10, 20, 50, and 100 μm in regions P5, P10, P20, P50, and P100, respectively. In all regions the gap between stripes (g) was equal to the stripe width (g = w). Stripe height (h) was 4 μm in standard samples and 1 μm in all regions in the testing sample. The flat area named “Ref” of 1000 µm wide, without stripes, was made as a reference. Each region P has dimensions 1.5 × 8 mm and typically is fabricated along the m-direction.

We used freestanding, c-plane (0001) GaN substrates from Saint-Gobain Lumilog company (Vallauris, France). Dislocation density was about 10^7^/cm^2^ and the off-cut angle was 0.6 ± 0.05° towards the m-direction <1–100>. Laser-beam photolithography (Microtech LW405B, Palermo, Italy) and ion etching with chlorine chemistry (Oxford Plasma Lab 100, Yatton, UK) were used to define the substrate pattern. The substrate scheme is shown in Figure 1a,b.

All prepared GaN substrates (11 × 14 mm in size) had five patterned regions marked P5, P10, P20, P50, and P100, each with size 1.5 × 8 mm. Stripe width, *w*, was 5, 10, 20, 50, and 100 µm in regions P5, P10, P20, P50, and P100, respectively, as shown in Figure 1a,b. In each region, stripe separation (g) was equal to the respective stripe width (*w*), as shown in Figure 1a. Between regions P50 and P100 there was a flat 1000 µm wide reference region without any stripes. Stripe direction was chosen along the m-direction <1–100> as to be sure that during the epitaxial growth atomic steps could flow along the top of the stripe. However, we also prepared substrate with stripes perpendicular to the m-direction, as shown in Table 1. To check the morphology of QWs grown this way, this sample will be described in Section 3.1. Typically, the pattern consisted of a set of 4 µm high parallel stripes. However, to check how luminescence depends on the height of the stripes we also prepared substrate with 1 µm high stripes, as shown in Table 1, and results are described in Section 3.5. Before epitaxy, all substrates were cleaned in organic solvents and piranha.

### 2.2. Epitaxy Method

The sidewall angle of patterned substrates before the growth was about 70–80° with respect to the (0001) plane. Then, InGaN/GaN structures were grown on them, using metalorganic vapor phase epitaxy (MOVPE). The growth processes were carried out on two MOVPE reactors—one of them was a home-built, vertical reactor [30] located in the Institute of High Pressure Physics, Warsaw, Poland (IHPP) and the other was a commercial Taiyo Nippon-Sanso horizontal reactor [31] located in the Amano Lab, Institute of Materials and Systems for Sustainability, Nagoya University, Nagoya, Japan (IMaSS). We used two different reactors to confirm that the observed effect was not specific to a particular epitaxy system. The epitaxial structure grown in the above mentioned reactors consisted of subsequent layers: 0.5 µm GaN:Si (growth temperature T_gr_ = 1040 °C), 170 nm undoped In_0.03_Ga_0.95_N buffer layer (T_gr_ = 810 °C), and a single pair of undoped, 2 nm thick In_0.25_Ga_0.75_N quantum well (QW)/8 nm GaN quantum barrier (QB) (T_gr_ = 760 °C). An identical structure was grown on substrates with stripes in both directions, i.e., parallel and perpendicular to the m-direction (see Section 3.1). For the purpose of HR-XRD examination, a similar structure was grown on substrates with stripes parallel to the m-direction using the vertical reactor. It contained 5 QB/QW pairs (thicknesses 2 and 8 nm, respectively) instead of one to improve the ability to track QW properties with X-ray methods—results are described in Section 3.3.

Efforts were made to carefully tune growth recipes for both reactors, to obtain identical structures. However, in MOVPE, indium composition is always very sensitive to many parameters, mainly growth temperature. For this reason, average indium composition achieved in the horizontal reactor was lower than for the vertical one. Nevertheless, the main effect was observed for structures grown in both reactors.

### 2.3. Sample Characterization

Morphology of samples was checked by tapping mode atomic force microscope (AFM, Veeco Dimension 3100, Plainview, NY, USA).

Optical properties were studied at room temperature using two methods—micro-photoluminescence (µPL) and cathodoluminescence (CL). In the µPL setup, luminescence was excited by a Kimmon He-Cd, continuous wave, 325 nm laser with 15 mW output power, and spectra were acquired by a iHR 320 spectrometer (Horiba Scientific, Piscataway, NJ, USA). Excitation spot size in this system was about 5 µm. CL measurements were performed with a SU-70 scanning electron microscope (Hitachi High Technologies, Tokyo, Japan) equipped with the Gatan Mono CL3 system (Pleasanton, CA, USA). Accelerating voltage ranged from 5 to 15 kV and the beam current from 2.4 to 14 nA.

Structural properties were evaluated on the sample with 5 QW/QB pairs to increase signal intensity from QWs/QBs and improve ability to calculate indium content and width of QWs and QBs. The HR-XRD system (Empyrean-Malvern Panalytical, Almelo, The Netherlands) with a CuKα_1_ X-ray source and equipped with hybrid 2-bounce monochromator and a threefold Ge (220) analyzer was used.

The structure grown in the horizontal reactor was investigated by scanning transmission electron microscopy (STEM) in high-angle annular dark-field (HAADF) operation mode, using Tecnai G2 F20 S-TWIN microscope (FEI Company, Hillsboro, OR, USA) operated at 200 kV. A cross-sectional STEM specimen was prepared by mechanical polishing and subsequent Ar+ final ion milling until electron transparency using a Veraion precision ion polishing system (Gatan, Pleasanton, CA, USA).

Additionally, we examined the structure grown on patterned substrate with 4 μm high stripes with a Dektak 150 stylus profiler (Veeco, Plainview, NY, USA).

## 3. Experimental Results

### 3.1. Influence of Stripe Azimuth on the InGaN Morphology

**Figure 2:**
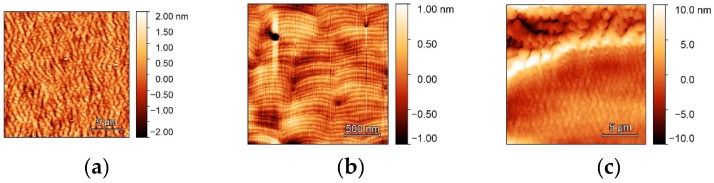
Atomic force microscope (AFM) height images of an InGaN/GaN structure grown on two substrates with stripes. In (**a**,**b**), stripe direction was parallel to substrate miscut azimuth and in (**c**) it was perpendicular.

All structures were grown in the step-flow mode. The structure morphology on substrates with stripes parallel to the m-direction were similar to each other, both grown using vertical and horizontal MOVPE reactors. AFM images for structure grown using a vertical reactor is shown in Figure 2a,b. The morphology of the same structure grown on substrate with stripes perpendicular to the m-direction, i.e., along a-direction, was different and is shown in Figure 2c.

The surface roughness (RMS) of QW grown on stripes parallel to the m–direction and measured by AFM on a 20 × 20 µm area was 0.52 nm while RMS of the QW grown on substrate with stripes perpendicular to the m-direction was 3.2 nm.

These differences in layer morphology correspond to the directions of the stripe with respect to the atomic step direction of the substrate. For the substrates with stripes parallel to the m-direction, atomic steps were perpendicular to the stripe boundary and during growth they could flow along the stripe top without disturbances, resulting in a smooth surface, as shown in Figure 2a,b. On the contrary, for the substrate with stripes perpendicular to the m-direction, atomic steps were parallel to the stripe boundary and during growth they flew across the stripe, as shown in Figure 2c. Propagation length of an atomic step during growth, *l*, can be estimated as *l* = *d*/tan*δ* where *d* is layer thickness and *δ* is the substrate miscut. In our case, *d* = 0.68 µm and *δ* = 0.6°, so *l* = 65 µm which is similar or larger than the stripe width. As a result, a large portion of the layer grew in an island mode due to the lack of source of atomic steps. The morphology of the InGaN QWs in the case of the stripes perpendicular to the m-direction was not acceptable due to large and non-uniform roughness; all further results are only for the QWs grown on substrates with stripes parallel to the m-direction.

### 3.2. Influence of Stripe width on Luminescence of InGaN QW

**Figure 3:**
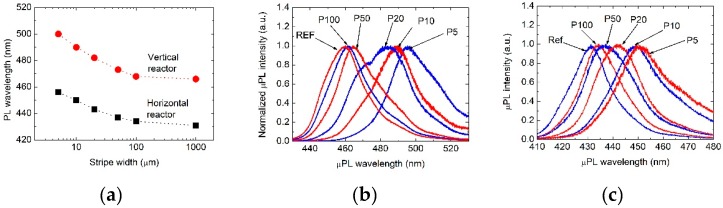
Micro-photoluminescence (µPL) of nominally identical InGaN/GaN structures grown on substrates with 4 μm high stripes using vertical and horizontal reactors. (**a**) Central wavelength of µPL as a function of stripe width, (**b**) normalized µPL spectra of structure grown using a vertical reactor, (**c**) normalized µPL spectra of structure grown using a horizontal reactor.

The most important result in the present paper is that luminescence wavelength of an InGaN QW/QB structure strongly increased when it was grown on a narrower stripe. More precisely, for single QW grown using a vertical reactor, µPL wavelength shifted from 461 up to 495 nm, and for stripe width variation, from 100 down to 5 µm, respectively. For nominally the same structure grown using a horizontal reactor, a similar effect was found, and µPL wavelength increased from 434 up to 451 nm, and for stripe width change, from 100 to 5 µm, respectively. In both cases µPL was measured on top of each stripe, in the middle between stripe edges at room temperature. Luminescence wavelength as a function of stripe width is shown in Figure 3a and µPL spectra are shown in Figure 3b,c.

**Figure 4:**
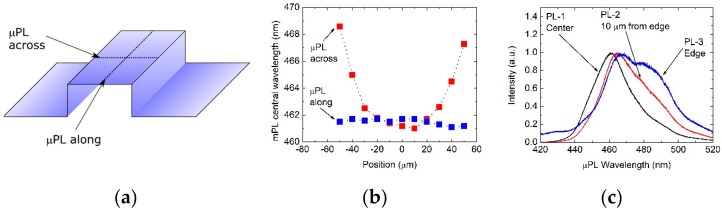
μPL of the InGaN/GaN structure. Stripe width 100 µm, stripe height 4 μm. (**a**) Measurement scheme with two scan lines across and along the stripe. (**b**) Wavelength across and along the stripe. (**c**) Spectra acquired at stripe center, 10 µm from stripe edge, and at the stripe edge.

**Figure 5:**
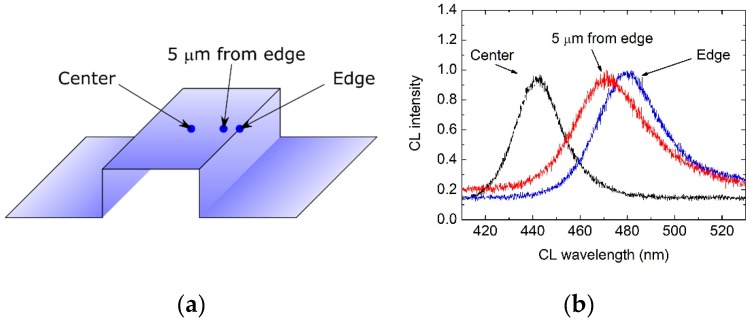
Cathodoluminescence (CL) of the InGaN/GaN structure. Stripe width was 50 µm. (**a**) Scheme of CL measurement with three characteristic points. (**b**) CL spectra acquired at stripe center, 5 µm from the stripe edge, and at the stripe edge.

In the next step we studied optical properties of our structure with single QW grown using a vertical, and also a horizontal reactor, in more detail. For both samples we performed µPL scans along and across stripes in all regions P5–P100, as shown in Figure 4a. We also made CL measurements in three spots on top of the 50 µm wide stripe—in the center, 5 µm from the edge, and at the edge, as shown in Figure 5a. The μPL measurement scheme and results (for 100 µm wide stripe) are shown in Figure 4. The CL measurement scheme and results (for 50 µm wide stripe) are shown in Figure 4. It turned out that central µPL wavelength is constant along the stripe. However, central luminescence wavelength and full-width at half maximum (FWHM) of µPL and CL spectra increased from the center of each stripe towards its edges.

### 3.3. Structural Properties

**Figure 6:**
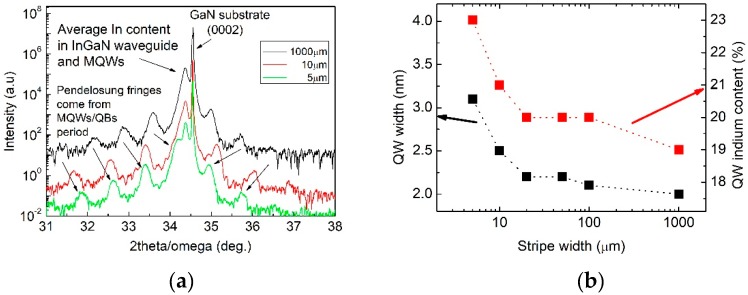
HR-XRD measurements of the InGaN/GaN MQW structure on substrate with 4 μm high stripes; (**a**) 2theta-omega scans collected at 1000 µm wide reference area, at 10 µm, and 5 µm stripe regions (other regions not shown for picture clarity); (**b**) quantum well (QW) width and QW indium composition for different stripe widths obtained by fitting respective HR-XRD scans. The X-ray beam covers the whole width of each stripe.

Structure parameters, i.e., QW and QB thicknesses and QW indium content were examined independently in each of the patterned regions P5–P100 and in the reference region (see Figure 1 for a region layout explanation). Parameter values were obtained using Panalytical Epitaxy software with implemented dynamical theory of diffraction, by fitting calculated X-ray scans to measured ones, as shown in Figure 6a. It was found that QW thickness increased from 2 up to 3 nm when stripe width was decreased from 100 down to 5 µm, respectively, as shown in Figure 6b. Changes of QW/QB thickness is clearly seen in Figure 6a by shifts of the Pendelosung fringes towards (0002) reflection of GaN substrate. Similarly, QB thickness increased slightly from 8.2 up to 9 nm. At the same time, indium composition in QWs increased from 19% on 100 µm wide stripes up to 23% on 5 µm wide stripes, as shown in Figure 6b. Of course, due to the composition pulling effect [32], on narrow stripes, the 1-QW and the 5-QW structures could differ. However, the observed wavelength variations with respect to the stripe width were similar for both structures.

### 3.4. Influence of TMIn Flow and Temperature on Growth Rate on the Stripes

**Figure 7:**
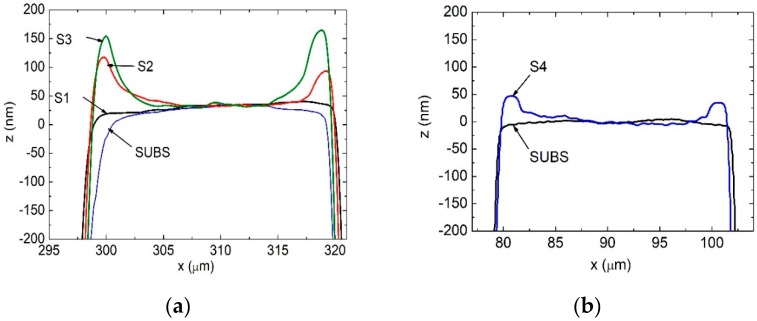
Surface profiles of InGaN/GaN structures grown on substrates with 20 µm wide stripes. All profiles have been shifted in the z-direction to match each other’s height in the center of the stripe. In (**a**) profile SUBS is for GaN substrate, S1—after growth of HT-GaN, S2—after HT-GaN and InGaN, S3 after HT-GaN, InGaN, and 5QWs. In (**b**) profile SUBS is for GaN substrate and S4 after growth of HT-GaN + LT-GaN. See text and Table 2 for more details.

To get more detail on the growth modes on the stripes, we examined the structure grown on patterned substrate with 4 μm high stripes with a stylus profiler. We scanned all patterned regions of the structure in a direction perpendicular to the stripes, i.e., across the stripes, as shown in Figure 7a. It turned out that the structure height in the vicinity of the stripe edges is 100–150 nm larger than in the stripe center.

Therefore, we decided to split our examined InGaN/GaN structure into parts, grow each of them separately on new patterned substrates, as shown in Table 2, and then check surface profile for each growth. Firstly, we measured the surface of patterned substrate in an identical manner (sample SUBS). Another profile was measured after growth of 0.5 µm high-temperature GaN:Si (HT-GaN, T_gr_ = 1040 °C, sample S1, see Table 2). Another profile was measured on epitaxial structure with 0.5 µm HT-GaN GaN, followed by 170 nm InGaN, T_gr_ = 810 °C, sample S2—see Table 2. The next profile was measured on epitaxial structure with five QW/QB pairs, sample S3—see Table 2. The results are shown in Figure 7a. Finally, we compared measurement of patterned substrate (sample SUBS), as shown in Table 2, to the sample which consisted of 0.5 µm HT-GaN:Si followed by 170 nm thick LT-GaN (low temperature GaN, T_gr_ = 810 °C), sample S4, Table 2. The results are shown in Figure 7b.

It turned out that the initial angle of the stripe sidewalls (70–80° to the c-plane) changed after growth of HT-GaN, because the exact-oriented, (11–22) facet has formed. An identical effect was observed in both reactors. That is why surface profiles of SUBS and S1 differ. Taking this fact into account, it can be seen that structure height at the stripe edges was larger only for GaN and InGaN layers grown at relatively low temperature, i.e., 810 °C or lower. For HT-GaN, structure height on top of the stripe was the same as for the patterned substrate without any grown structure.

From HR-XRD results we learnt that the thickness of InGaN buffer, QWs, and QBs grown on top of the stripe were not lower but rather greater than the thickness of the same layers grown on the flat reference region. Nominally, the InGaN buffer thickness was 170 nm. However, the height of the stripe measured near the stripe’s edge is 100 nm larger than the height measured near the stripe’s center. This means that the growth rate of the InGaN buffer close to the stripe edge must have been 60% larger than in the stripe center. Nominal thickness of five pairs of QW/QB was 50 nm. Their surface profile close to the stripe edge extends by ~50 nm over the InGaN buffer. It means that MQWs must have grown 100% faster close to the stripe edge compared to the center.

### 3.5. Influence of the Stripe Height on Luminescence of InGaN QWs.

**Figure 8:**
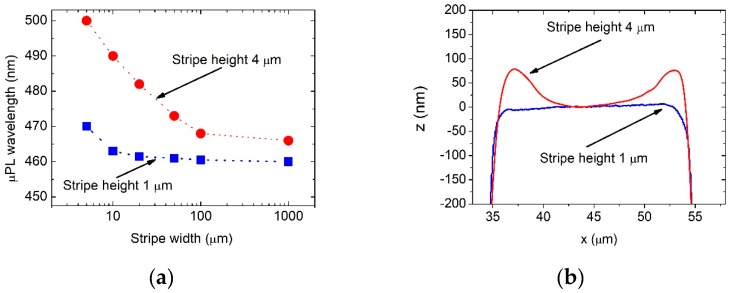
Luminescence (**a**) and surface profiles (**b**) of QW grown on substrates with stripes of different height.

To investigate the influence of stripe height on morphology and optical properties of InGaN/GaN structures, we prepared another patterned substrate. It was nearly identical to the substrate described in Figure 1, but the stripe height was only 1 µm instead of 4 µm. On this substrate we grew an InGaN/GaN epitaxial structure, identical to the one described in Section 2.2. It turned out that the luminescence wavelength shift in the case of 1 µm high stripes was only 10 nm between the reference region and 5 µm wide stripes, as shown in Figure 8a. Moreover, the effect of faster growth near the stripe edges was not observed for 1 µm high stripes, as shown in Figure 8b.

## 4. Discussion

The observed luminescence wavelength variations on different stripe widths may depend on the following factors and their combinations: (i) variations in quantum-confined Stark effect (QCSE) due to strain, (ii) variations in QW thickness, and (iii) variations in QW indium composition.

**Figure 9:**
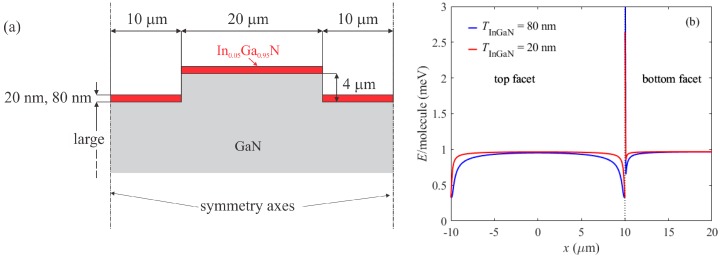
(**a**) Structure model used for the calculation of elastic energy. (**b**) Lateral profiles of the elastic energy per one InGaN molecule at the top and bottom InGaN surfaces.

In order to assess the influence of elastic strains we performed numerical simulations of elastic energy per one InGaN molecule at the free surfaces of the stripe and the trench between the stripes, and the results are presented in Figure 9. For the simulations we used a standard finite-element method, and elastic constants of GaN and InGaN were taken from [13,33]. We performed strain simulations for the 20 µm wide stripe and it turned out that in our design of patterned substrate, the influence of strain was limited to 2–3 µm from the stripe edge, as shown in Figure 9. However, from µPL data we see that luminescence wavelength shifts were observed not only near the stripe edges but on the whole 20 µm wide stripe (wavelength shifts more than 20 nm at the stripe center, compared to the flat region of the substrate). Therefore, we conclude that mechanical strain could have an influence on wavelength shift only near the stripe edge and is probably not the main cause for the observed phenomena.

Next, we tried to correlate QW thickness, growth rate, and indium composition on different stripes. According to HR-XRD measurements in the present work (Section 3.3), made on the 5QW structure, thicknesses of QWs and QBs on 5 µm wide stripes were 3 and 9 nm, respectively. However, on 100 µm wide stripes the thicknesses were only 2 and 8 nm, respectively. Indium composition in QWs was 23% for the 5 µm stripe and 19% for the 100 µm one, as shown in Figure 6. Thus, the growth rate of the QW was 0.33 Å/s on 100 µm stripes and 0.5 Å/s for 5 µm ones.

The influence of the growth rate on indium composition has been investigated by Leszczyński et al. [29]. In that work, it was explained that incorporation of indium is larger at the higher growth rate because it is necessary to overbuild the indium atoms by gallium, otherwise it desorbs from the surface. Increased indium composition on narrow stripes could have been solely the result of faster growth. Hence, the increased µPL wavelength observed on narrow stripes can be explained by the joint effect of increased indium content and thicker QWs (thicker wells emit in longer wavelengths due to stronger QCSE).

Finally, we would like to know why the growth rate is higher on narrow stripes. As the observed effects occurred during MOVPE growth on patterned substrates, they should be discussed taking into account the following phenomena: (i) gas phase transport [34,35,36], (ii) gas phase reactions [37,38,39], (iii) gas phase diffusion [40,41,42,43,44,45], and (iv) surface diffusion [46,47,48,49].

Gas phase transport depends on the reactor design details. However, since we observed the effect in two very different reactors, we conclude that gas phase transport is not the most important factor here and we neglect it.

Concerning gas phase reactions and gas phase diffusion, the presence of stripes (uneven surface) influences decomposition of the active species, their diffusion, and incorporation. The observed faster growth at the stripe edge would suggest that locally (edge area) the density of the active species participating in the growth is higher than in the stripe center or on the flat reference region. Because in our experiments the III-element sources were trimethylgallium and trimethylindium, the gas phase was dominated by their respective trimethyl (TM) and dimethyl (DM) species—DM resulting from TM decomposition. Although the DM lifetime is extremely short, it decomposes into monomethyl (MM), which diffuses on the surface with a given diffusion length, and in turn, is decomposed in adatoms, which also diffuse on the surface with their own diffusion lengths. All these species are affected by the presence of the stripes. The gas phase was preferentially decomposed at the stripe edges (possibly because of a higher temperature gradient [50]), thus inducing higher density of species (MM and adatoms) diffusing on the surface. As the stripe is higher, this effect becomes more pronounced.

Surface diffusion of MM species and adatoms will affect observed phenomena in a different way. The MM influences the distribution of adatoms on the surface, which consequently influences the local growth rate. Diffusion lengths of MM and adatoms can be estimated from the present experimental results. In this view, different indium compositions on different stripes, as shown in Figure 6b, would be an effect of MMIn diffusion (InGaN growth being In-limited), which is probably between 5 and 10 µm, since the composition was nearly constant for stripe widths larger than 20 µm. Similarly, diffusion of In adatoms is probably around 3–3.5 µm and it would explain faster growth observed up to 6–7 µm from the stripes’ edge, as shown in Figure 7. Thus, the adatom’s diffusion explains the overgrowth at the stripe edge, and the diffusion of MM species explains why the concentration of indium is higher on small stripes.

On the other hand, such explanation is based on experiments where selective masks were used, and this is not the case of the present work. Therefore, we would like to present other arguments too.

We take into account the following effects: (i) gas-phase diffusion, (ii) alloy-pulling effect of indium due to mechanical strain, and (iii) kinetic processes on the surface. It ’is important to note that the faster growth at the edges is present only when growth temperature is relatively low (820 °C rather than 1000 °C) and when the stripe height is relatively large (4 µm rather than 1 µm). Moreover, the effect is two times stronger for LT InGaN than for LT-GaN grown at the same conditions. There is no effect for structures grown at 1000 °C, even on 4 µm high stripes, and there is no effect for structures grown on 1 µm high stripes even at 820 °C.

Certainly, gas phase diffusion would promote faster growth at the stripe edge. In the general case, based on a numerical solution of the diffusion equation, the distance which adatoms have to travel through the gas before they can adsorb at the crystal surface is the smallest at the edges, as in [40,51], as shown in Figure 8. Our own results obtained by a simple 2D, Monte Carlo simulation of the diffusion process confirms this effect.

Another point is that the faster growth of LT-InGaN compared to LT-GaN could be explained by the reduced alloy-pulling effect for indium at the 2–3 µm wide area close to the stripe edge [32]. Partial elastic relaxation of the structure near the edges would account for the low energy barrier for indium adatoms to incorporate into the crystal, as shown in Figure 9.

**Figure 10:**
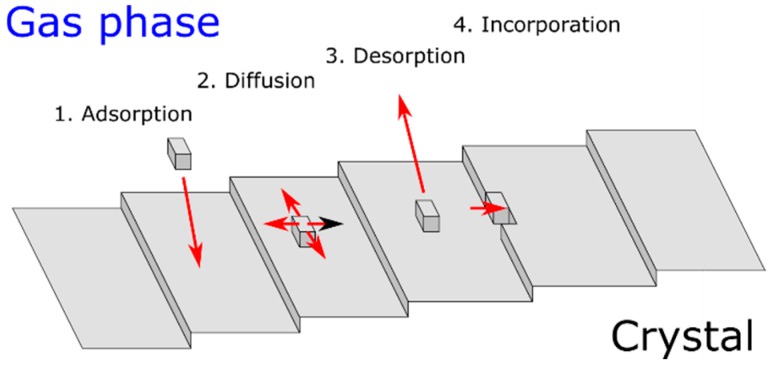
Different processes taken into account in the BCF model of crystal growth on vicinal surfaces.

However, the most important here are kinetic processes at the growth surface. First, based on AFM data, we point out that the structure grows in the step-flow mode and thus the growth could be described in the framework of the Burton-Cabrera-Frank model [52], as shown in Figure 10. In this model adatoms adsorb on the growth surface from the gas phase and then diffuse, before they are caught by atomic steps and incorporate into the crystal. The second possibility is, however, that they desorb and return to the gas phase. The latter can happen mainly in high temperatures and low miscut conditions, i.e., when the atomic terrace width is comparable or larger than the adatom’s surface diffusion length.

**Figure 11:**
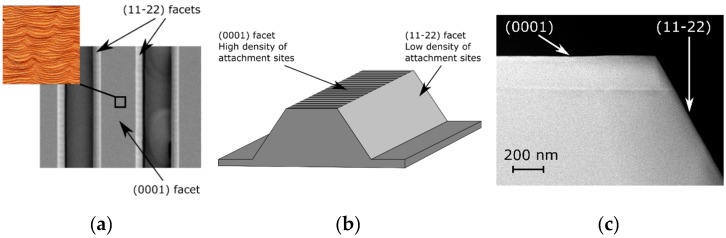
**(a**) Top-view SEM image of the structure grown on substrate with stripes. Image size 40 × 30 µm. Inset: 2 × 2 µm AFM image acquired on the top of a 10 µm wide stripe. (**b**) Schematic perspective view of the structure. The top of the stripe is formed of the vicinal (0001) plane and the sidewalls are exact-oriented (11–22) planes. (**c**) Cross-sectional scanning transmission electron microscopy (STEM) image.

Second, it is important to look at the structure morphology after growth. Judging from AFM, SEM, and STEM data, the top of the stripe is formed of (0001) plane which preserves the initial miscut of the substrate (0.6° towards the <1–100> direction), i.e., it contains regular atomic steps, with the average direction perpendicular to the stripe edge, separated by 20–30 nm wide terraces, as shown in Figure 2b and inset to Figure 11a. On the contrary, the stripes’ direction was precisely aligned 90 ± 0.1° to the easy-cleavage direction of GaN, <11–20>, and the sidewalls of the stripe (which are about 6 µm wide for a 4 µm stripe height) after growth become exact-oriented, low-index (11–22) planes, without any steps, as shown in Figure 11b,c. Hence, the density of sites where adatoms can attach is relatively high on the top of the stripe, compared to the sidewalls, where there are almost no such sites. It is important to note that on the (11–22) facet there was almost no growth, as shown in Figure 11c.

Now let ’us assume that adatoms from the gas phase adsorb to both mentioned surfaces, i.e., the (0001) and (11–22) at equal rates. After adsorption they start to diffuse randomly on the surfaces. In the BCF theory, mean diffusion length of adatoms on the growth surface is expressed as

λ = a exp [(W_s_ − U_s_)/2kT] (1)

where: a—lattice parameter in the growth plane, U_s_—energy barrier for adatom to move to the next stable position (process “2” from Figure 11), W_s_—energy barrier for adatom desorption (process “3” from Figure 10), T—absolute temperature.

Assuming that the growth is performed in nitrogen-rich conditions (which is almost always true in the case of MOVPE), growth kinetics will be governed by Ga and In diffusion on the surface. Mean diffusion lengths of these adatoms on (0001) and (11–22) facets have been estimated by Ueda and coworkers [42]. Those authors investigated InGaN grown by MOVPE on patterned substrate with a SiO_2_ mask. The growth temperature was not specified but we assume that it must have been adequate for InGaN growth, i.e., about 800 °C. On (0001) they were 5.2 and 2.7 µm for Ga and In, respectively, and on (11–22) they were 3.1 and 1.6 µm for Ga and In, respectively. Hence, adatoms caught on the stripe top easily find an atomic step and attach (distance between adjacent atomic steps is 20–30 nm there). On the other hand, adatoms caught on the stripe sidewalls continue to diffuse until they reach the stripe edge because the surface is atomically flat and there are no atomic steps. Eventually they desorb back to the gas phase.

The balance between the caught and desorbed number of adatoms will depend on their mean diffusion length, which drops rapidly with temperature. At typical temperatures used for InGaN growth, the diffusion length is a few micrometers, i.e., of the order of the sidewall width [42]. Hence the (11–22) facets will catch adatoms and direct them towards the (0001) top facet where they can incorporate. The conclusion here is that the larger diffusion length, the faster growth near the stripe edge, i.e., fast growth will be promoted for low growth temperature. Faster growth at the stripe edges will also be promoted on taller stripes, because then the sidewall surface is larger. Since the (11–22) facet angle to the (0001) plane is 58.4 degrees [53], the (11–22) sidewall width = 1.6 times the stripe height. This was confirmed by our results on 1 and 4µm tall stripes and by surface profiles of GaN grown at 800 and 1000 °C.

To sum up, faster growth of InGaN and its increased indium composition at the stripe edge could have been caused by all three factors—gas phase diffusion, the alloy-pulling effect, and kinetic processes—and the last factor is probably the most important.

## 5. Conclusions

GaN and InGaN layers, and InGaN/GaN MQW structures were grown on (0001) GaN substrates with stripes, at different growth temperatures, by MOVPE. The substrate stripes were parallel to the <1–100> direction, they were 5–100 µm wide, and typically 4 µm high (1 µm on the test sample). There was no selective mask on the substrate. It was found that central wavelength of luminescence of MQW structures depend on the stripe width and height, and also growth temperature. It can be up to 40 nm larger on 5 µm wide, 4 µm tall stripes, compared to the flat area of the substrate (QW growth temperature 760 °C). We attribute this effect to faster growth on tall and narrow stripes. Faster growth promotes more effective indium incorporation, and of course QWs are thicker, which both account for the observed effect. Faster growth on tall and narrow stripes is caused mainly by kinetic processes at the growth surface. Notably, the initial substrate miscut angle and azimuth, and their relation to the direction of stripes is of primary importance for the observed effects. The reduced alloy-pulling effect for indium, due to partial elastic strain relaxation at the stripe edges, could also add to the observed effect.

## Figures and Tables

**Table 1:** List of substrates. Symbols ∥ and ⟂ mean “parallel to” and “perpendicular to”, respectively.

Substrate	d (µm)	Azimuth 3
1	4	∥ m
2	4	⟂ m
3	1	∥ m

**Table 2:** Summary of samples used for surface profile characterization. QB: quantum barrier.

Sample Name	Epitaxial Structure	Growth Temperature (°C)
SUBS	Patterned substrate	
S1	HT-GaN	1040
S2	HT-GaN + InGaN buffer	1040 + 810
S3	GaN + InGaN buffer + 5 × (QW + QB)	1040 + 810 + 760
S4	HT-GaN + LT-GaN	1040 + 810
